# The redistributive effects of copayment in outpatient prescriptions: evidence from Lombardy

**DOI:** 10.1186/s12913-017-2248-6

**Published:** 2017-05-08

**Authors:** Paolo Berta, Rosella Levaggi, Gianmaria Martini, Stefano Verzillo

**Affiliations:** 1 Department of Quantitative Methods, University of Milan Bicocca, Via Bicocca degli Arcimboldi, 8, Milan, 20146 Italy; 2 Department of Economics and Management, University of Brescia, Via San Faustino 74b, Brescia, 25100 Italy; 3 Department of Management, Information and Production Engineering, University of Bergamo, Via Pasubio 7/b, Dalmine, 24044 Italy; 4 Department of Economics, Management and Quantitative Methods, University of Milan, Via Conservatorio, 7, Milan, 20126 Italy; 5 European Commission, Joint Research Centre, Via E. Fermi 2749, TP 361, Ispra, 21027 (VA) Italy; 6 CRISP, University of Milan-Bicocca, Via Bicocca degli Arcimboldi, 8, Milan, 20126 Italy

**Keywords:** Copayment, Superticket, Redistributive effects, Lombardy

## Abstract

**Background:**

In Italy, copayment has changed its nature and it can no longer be simply considered a system to curb inappropriate expenditure. It has become an important form of revenue for public health care provision, but it might also become a source of distortions in income and health benefits redistribution.

**Methods:**

We use a rich administrative dataset gathering information on patients demand (whose records have been matched to income declared for tax purposes) to study the effects of an additional copayment (the so called “superticket” introduced by the Italian government in 2012) in Lombardy, the biggest Italian Region whose socio-economic dimension is comparable to that of many European countries (e.g., the Netherlands, Switzerland, etc.).

**Results:**

Our analysis shows that at the aggregate level the non-uniform superticket schedule adopted in Lombardy is slightly pro-poor, but this result coexists with evidences pointing towards possible cases of restriction to access caused by the additional copayment.

**Conclusions:**

The introduction of the superticket and the ensuing increase in the out-of pocket payment for health care raises questions about the distribution of the burden among patients, and the sustainability of the extra revenue through time. This issue needs to be further investigated by combining health status data with the information in this dataset.

## Background

Copayment was firstly introduced in public health care systems to curb inappropriate expenditure; nowadays it produces substantial revenues, but it might become a barrier to access to health care [1, 2, 4, 5, 9]. In this article we focus on the effects of the increase in the copayment for diagnostic tests and ambulatory care in Italy where copayments are means and health tested through a set of exemptions set at national and regional level [7, 10].

Since 2012, two different cost sharing schemes coexist: a regional and a national one (the so called “superticket”). In that year the Italian Government reduced the equalisation grant to each Region by an amount equal to €10 times the number of prescriptions reimbursed by each Regional Health care System (RHS) in 2011. Regions were allowed to set their own superticket schedule to cover the gap [7, 10]. Three schemes were adopted: **(1)** a uniform superticket equal to €10 for each prescription; **(2)** a cost-related extra payment and **(3)** a means tested superticket.

In this paper we analyse the effects of the introduction of the superticket on outpatient prescriptions in Lombardy using a rich administrative dataset with information on patients demand whose records have been matched to income declared for tax purposes. Lombardy is the biggest Italian region, with a population of about 10 million inhabitants (17% of Italy’s population) and a GDP equal to 25% of the national one. Hence, its socio-economic dimension is comparable with that of many European countries (e.g., the Netherlands, Switzerland, etc.).

For the superticket, Lombardy adopted a cost-related scheme with an extra-payment proportional to the cost of service/reimbursement schedule, starting from €0 (for treatments whose regional reimbursement is below €5) to €30 (for prescriptions with reimbursement above €100), as shown in Table 1 (column #2). The schedule was determined by the Regional Government using a budget balance hypothesis. Taking year 2011 as reference, and under the assumption of no change in the demand composition, the superticket in 2012 should have exactly compensated the reduction in the national equalisation grant, i.e., about 135 million Euro.

**Table 1:** Copayment, superticket, prescriptions, revenue and cost for regional health service. Lombardy, 2012

Cost of	Regional	Superticket	Number of	Cost for	Regional Ticket	Superticket	Relative
service (€)	Copayment (€)	(€)	prescriptions	RHS (€)	revenue (€)	revenue (€)	price
<5	Cost of service	0	1,251,256	3,922,183.5	1,280,274.2	20	0.33
5.01–10	Cost of service	1.5	766,241	5,776,744.0	5,766,124.4	1,152,325	1.20
10.1–15	Cost of service	3	972,005	12,266,593.2	12,199,577.8	2,902,677.8	1.23
15.01–20	Cost of service	4.5	2,851,658	49,988,059.1	49,819,215.9	12,783,255.1	1.25
20.01–25	Cost of service	6	2,532,925	56,989,625.0	56,767,377.5	15,119,559.4	1.26
25.01–30	Cost of service	7.5	636,307	17,510,193.5	17,099,649.5	4,644,582.1	1.24
30.01–36	Cost of service	9	977,603	31,970,282.8	31,464,748.6	8,646,470.1	1.25
36.01–41	36	10.8	480,011	18,734,049.8	16,893,854.7	5,055,352.5	1.17
41.01–46	36	12.3	619,499	27,294,168.8	22,026,288.1	7,503,356.8	1.08
46.01–51	36	13.8	219,843	10,573,257.9	7,726,886.7	2,933,118.7	1.01
51.01–56	36	15.3	286,473	15,113,567.4	10,154,370.6	4,278,278.2	0.95
56.01–65	36	16.8	458,551	27,984,658.1	16,348,194.7	7,572,823.6	0.85
65.01–76	36	19.5	515,575	36,303,131.1	18,480,666.2	9,952,636.2	0.78
76.01–85	36	22.8	283,236	23,142,023.3	10,165,653.3	6,396,458.7	0.72
85.01–100	36	25.5	210,611	19,537,531	7,563,370	5,322,495.1	0.66
>100	36	30	1,160,358	327,923,400.1	41,654,852.1	34,598,231.3	0.23
Total			14,222,152	685,029,469	325,411,105	128,861,641	0.66

The schedule adopted in Lombardy allows to reduce the burden of the extra charge on low value prescriptions, which would be priced well above their reimbursement cost, but it does not avoid overcharging completely. As a result, for outpatient prescriptions whose user charge is below €51 patients pay more than the amount reimbursed by Lombardy to the provider. For prescriptions above this threshold patients pay instead less than the cost (i.e., copayment is restored), but the superticket varies from €15 to €30.

In this paper we use a unique dataset to study the distribution of the revenue of the superticket across two dimensions: (1) the value of the prescriptions (defined as the amount paid to the provider by the Lombardy Region) and (2) the group of individuals that has to pay more (or less) for it. The first dimension allows to determine the actual distribution of the superticket across prescriptions while the second dimension allows some speculations on the distributive impact of this extra charge.

## Methods

**Fig. 1:**
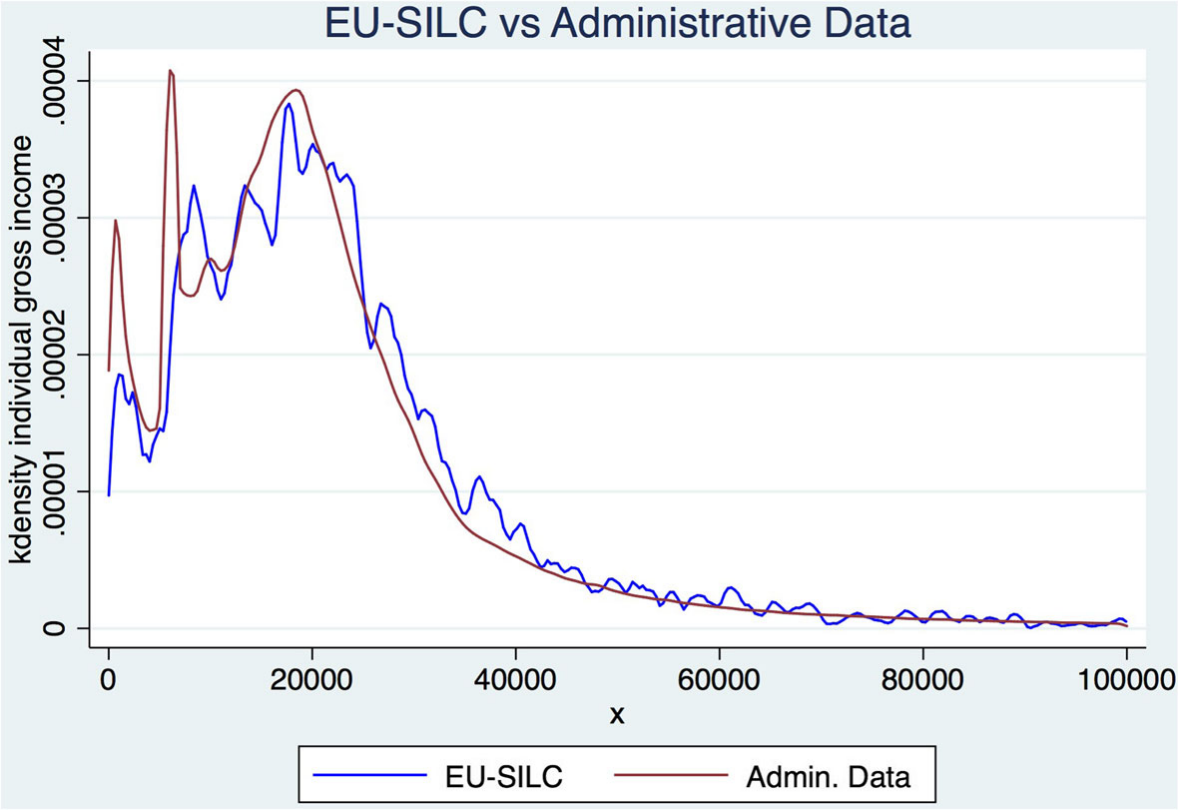
Individual income distribution

The dataset used includes individual administrative records provided by the Regional Social Health Care Information System on outpatients prescriptions and by the Tax and Income Department of the Lombardy Region on individual and family incomes. Data for about 9 million individuals with at least one outpatient prescription in 2012 were recorded and treated after being made anonymous. The outpatient database collects data for administrative purposes regarding 40,634,616 prescriptions (reporting the value of prescription, the regional copayment, the superticket, etc.). Individual and family incomes are related to year 2010 and were then associated to each citizen to investigate the relationship between disposable income and healthcare consumption levels (See [11] for a detailed overview of the system). Fiscal data may underestimate true income for self employed (due to omissions and tax evasion), but they represent the only reliable source of data at individual level. For this reason, we present our empirical analysis for employees and self-employed and check whether there is any significant difference in the relationship between income and prescriptions. Figure 1 compares the income distribution between our 2010 Lombardy income declaration data and the EU-SILC Lombardy income data.^1^ The two distributions are clearly similar with the only exception of a small difference in the left tie.^2^

Another possible shortcoming of our data is the two-year lag between individual income and outpatient consumption datasets. If it is possible that a small fraction of individuals^3^ may have experienced a reduction in their ability to pay for health care due to the global economic crisis (but we can control for this using income exemptions in 2012), it should also be noted that the timing of income declaration in Italy justifies the two-year lag between income and prescription data. In order to obtain a copayment (full or partial) exemption in 2012 individuals have to apply and show their latest income declaration. For this reason, in the period January-September 2012 the latest income declaration is that of 2011, related to 2010 income.^4^

The copayment system foresees two types of exemptions: income and chronic condition.^5^ In Lombardy, individuals (and their family members with no income) may be eligible for an income exemption (i.e., any prescription free of charge) if they fall into one of the following groups:

- the family income is below €8,263.31 (or €11,362.05 with a spouse with no income) increased by €516.46 for each children;
- the family income is below €36,151.98 and members are aged 65+;
- social pension (equal to €5,824.91) recipients;
- unemployed registered in employment centers.

Chronic patients suffering from a set list of illnesses are also eligible to receive free health care, provided that the prescription is related to their chronic condition. This information is available in our dataset and these individuals (exempted for income or chronic condition) have been excluded from the analysis; the dataset so obtained was then sorted according to the value of the prescriptions.

As shown in Table 1, prescriptions have been sorted in 17 groups which correspond to the superticket schedule foreseen by Lombardy; the lowest class corresponds to a provider’s reimbursement lower than €5 (and no superticket), while the highest has a prescription cost higher than €100 (and a superticket of €30). For each class we record the copayment (column #2), the superticket schedule (column #3), the total number of prescriptions (column #4), the total costs for the regional health system (column #5), the regional health system revenue (column #6), the superticket revenue (column #7) and its relative price (column #8). The latter is defined as the sum of copayment and superticket revenues divided by the total costs.

In 2012 the regional accounting system still allowed for “mixed prescriptions”, i.e., disease-specific exempt treatments that could be jointly prescribed with non-exempt ones. Unfortunately, information in the dataset does not allow to separate the prescription costs and revenues due to non-exempt treatments. As a consequence, the relative price presented in the last column of Table 1 may be underestimated. For the treatments falling into the €0-36 classes it is possible to identify these mixed prescriptions and compute their frequencies (see Table 2). Their contribution to the superticket revenue is only 0.52% in the €5.01-10 cost of service class and at most for 4.64% in the €25.01-30 class. We are confident that they do not alter the qualitative results of our analysis.

**Table 2:** Total, non exempt and “mixed” prescriptions. Lombardy, 2012

Cost of	Total	Non exempt	Mixed	Mixed	Superticket
Service (€)	prescriptions	prescriptions	prescriptions	(% on total)	
<5	1,251,256	2	1,251,254	0	99.99%
5.01-10	766,241	762,247	3,994	1.5	0.52%
10.1-15	972,005	958,760	13,245	3	1.36%
15.01-20	2,851,658	2,826,108	25,550	4.5	0.90%
20.01-25	2,532,925	2,510,297	22,628	6	0.89%
25.01-30	636,307	606,755	29,552	7.5	4.64%
30.01-36	977,603	946,667	30,936	9	3.16%

Our main goal is to identify the distributive impact of the superticket. We use three measures: (1) the Kakwany index; (2) the ANOVA analysis, and (3) the econometric analysis of individual demand and costs for the regional health service (based on income, demand groups and employment status). The Kakwani index *K* [3], is given by the following expression:

$$K=G_{S}-G_{I} $$

where *G*_*S*_ is the Gini concentration index of the extra payment due to superticket and *G*_*I*_ is the Gini coefficient for the income distribution. To evaluate *G*_*S*_ and *G*_*I*_, individual income and prescription expenditure were divided into seven groups of family income, as shown in Table 3. The first group (income lower than €8,000) represents the cut-off for income exemption. The other classes have an income varying from €8,000 to more than €75,000.

**Table 3:** Distribution of prescriptions and patients’ payments by demand type, Lombardy, 2012

Family	Exempt	Group #1			Group #2			Group #3		
income (€)	patients	Patients with relative price <1			Patients with relative price >1			Non exempt-high users		
	Number	Number	Average	Av. presc.	Number	Average	Av. presc.	Number	Average	Av. presc.
			pay (€)	number		pay (€)	number		pay (€)	number
<8,000	335,877	27,892	23.55	1.29	145,167	12.22	2.06	98,395	51.90	4.96
8,001-15,000	508,364	32,302	22.20	1.28	157,252	12.33	2.06	114,176	53.63	5.15
15,001-24,000	906,051	73,338	23.88	1.30	356,336	12.71	2.12	285,970	55.25	5.24
24,001-35,000	716,073	61,622	24.93	1.32	293,852	13.32	2.20	264,775	57.78	5.42
35,001-55,000	616,505	69,321	27.20	1.36	364,339	14.30	2.34	386,695	61.03	5.73
55,001-75,000	228,628	29,548	28.38	1.37	155,955	14.83	2.40	172,889	61.89	5.81
>75,001	215,432	35,329	29.66	1.37	161,780	14.75	2.38	179,125	60.85	5.66
Total	3,526,930	329,352	25.61	1.32	1,634,681	13.50	2.20	1,502,025	58.27	5.36
Not matched	585,151	40,103			210,264		141,277			

For each income category we divide users into three demand groups: Group #1, where patients demand only prescriptions whose cost is above €51 (i.e., with relative price < 1). Patients in this group may be charged a superticket varying between €15.01 and €30. For these patients the superticket is higher than what they would have paid under a uniform regime, but the treatments they demand are partially subsidized. Group #2 represents patients that have demanded only prescriptions whose cost is below €51 (relative price > 1). They benefit from the non uniform superticket regime schedule since the average superticket they pay is less than €10. However, their relative price is higher than 1 which implies had they addressed the demand to the private sector they could have been charged a lower price. Group #3 represents individuals that have paid the copayment for at least one prescription both with relative price below and above 1.

The ANOVA analysis is used to test whether the average superticket payment is significantly different among the 7 income classes in the three demand groups. An *F* test for the null hypothesis that the average superticket expenditure is the same allows to accept or reject this hypothesis.^6^

The demand for prescriptions has been further investigated using a cross-section analysis at individual level. The following model is estimated:

1 $$ Y=a+b\mathbf{X+}c\Psi+\epsilon  $$

where *Y* is the individual copayment expenditure (Model #1) or the cost generated by the demand (Model #2). **X** is a vector of individual characteristics (sex, age, marital status, number of children, number of disabled persons in the family, the local health authority of residence - *Azienda Sanitaria Locale*–ASL). **Ψ** is a vector of covariates that includes income class, the demand group (i.e., Group #1, #2 or #3), the employment status (employee, pensioner, self-employed) and some interaction variables.

Equation (1) has been estimated for the whole sample with the inclusion of a dummy variable for self-employed, and for a sub samples consisting of employees and self-employed only. In this way we can control for tax evasion.

## Results

In year 2012 the total number of prescriptions in Lombardy was equal to 40,634,616. The superticket was paid only for 12,970,896 prescriptions, since 27,789,625 prescriptions were exempt and 1,251,256 were in the €0-5 class, whose superticket is equal to 0 (see Table 1). The total revenue generated amounted to €132 million which is quite close to the target of €135 million that Lombardy had to reach in order to compensate for the lower grant received from the Central Government. The revenue generated by residents in Lombardy is equal to €128,861,641 (see Table 1) while the rest has been paid by non residents.

Prescriptions over €100 account for 26% of the revenue while those with cost range €15-25 raise about 20%. The rest is spread evenly among the other classes. The third column in Table 1 shows the superticket schedule. Prescriptions up to €36 benefit from the non uniform schedule adopted in Lombardy since they are charged a superticket lower than €10. They represent 9,987,995 prescriptions (70% of the total). As mentioned before, the last column in Table 1 shows the relative price in each class. The average relative price is 1.2, i.e. patients pay 20% more than the regional reimbursement. The cost to the Regional Health System for prescriptions falling in this range (where most of the demand is concentrated) is equal to €235,025,158; the revenue for the Regional Government is equal to €281,784,715. Hence, the net gain for the Lombardy Region is €46,759,557.^7^ In the range above €51 the relative price varies between 0.23 and 0.95 and the copayment regime is restored.

The average superticket is about €10; for a prescription whose cost is below €51 is equal to €6, while for those with cost higher than €51 is equal to €23.50, with a limited and not significant variance (standard deviation equal to €2.54) between income groups.

**Fig. 2:**
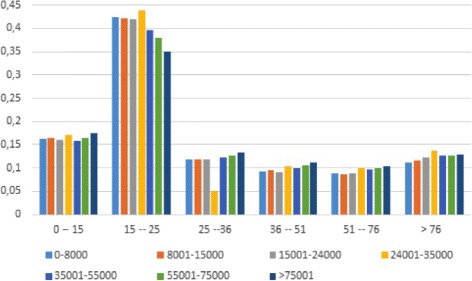
Distribution of prescriptions by income groups

Figure 2 shows the distribution of prescriptions among different income groups per cost of service class. About 35-40% of the prescriptions fall in the €15-25 cost class, about 15% belongs to the €0-15 class and the rest is evenly distributed among the other classes. The distribution is skewed to the right for low income groups, which means that the demand for costly prescriptions is more frequently made by high income individuals. Given the superticket schedule presented in Table 1 this means that its payment is borne more by rich than poor individuals. The Kakwani index is equal to 0.21: this is a first evidence that the effect of the superticket regime introduced in Lombardy is slightly progressive. Further insights into the distribution of the payment can be gained by analysing the superticket payments and consumption for the three previously identified demand groups: Group #1 (the high-cost consumption group), Group #2 (the low-cost consumption group) and Group #3 (the mix consumption group). The average superticket for patients belonging to Group #1 is €25.61 (see Table 3). The average payment is increasing in income, from €23.55 for the lowest income class to €29.66 for the highest one (the difference is on average €6.11, i.e. +25.9%).

The average superticket for patients in Group #2 is equal to €13.50, about half of the average payment in Group #1. Again, the average payment is slightly increasing in income (from €12.22 for patients belonging to the lowest income group to €14.75 for those in the highest one). On average, the highest income class pays €2.55 (+20.9%) more that the lowest class.

The picture changes dramatically when we examine Group #3 (the last two columns of Table 3). The annual average superticket shoots up to €58.27, with a significant difference between the bottom and the top end of the income distribution (€51.90 for the lowest income class and €60.85 for highest class, i.e, €8.95).

Table 3 also presents the average number of annual prescriptions by income classes among the three demand groups. The annual number of prescriptions is lower in Group #1 (1.32) and higher in Group #3 (5.36 more than 4 times higher), with Group #2 in the middle (2.20). For this reason, we can consider individuals in Group #3 as high users. The difference in average consumption between bottom and top income groups is +6.2% in Group #1, +15.5% in Group #2 and +14.1% in Group #3. Consumption is higher the greater is the income, and this is particularly important for consumers belonging to Group #3, the high users.

A more robust evidence of the effects of the superticket on health care demand may be obtained by applying the ANOVA analysis to test for the difference in expenditure and in the number in prescription across demand groups. The results are shown in Tables 4 and 5.

**Table 4:** Tests for differences in superticket payments among income classes

Group #1		
*F*–statistic	*P*-value	*H*_0_: equal mean among income classes
715.4	0.00 ^∗∗∗^	not accepted
Observations: 369,455		
Group #2		
*F*–statistic	*P*-value	*H*_0_: equal mean among income classes
2,046.4	0.00 ^∗∗∗^	not accepted
Observations: 1,844,945		
Group #3		
*F*–statistic	*P*-value	*H*_0_: equal mean among income classes
1,604.	0.00 ^∗∗∗^	not accepted
Observations: 1,643,302		

Legend: ^∗∗∗^ = 1% statistical significance

**Table 5:** Tests for differences in number of prescriptions among income classes

Group #1		
*F*–statistic	*P*-value	*H*_0_: equal mean among income classes
13.022	0.00 ^∗∗∗^	not accepted
Observations: 369,455		
Group #2		
*F*–statistic	*P*-value	*H*_0_: equal mean among income classes
2,929.995	0.00 ^∗∗∗^	not accepted
Observations: 1,844,945		
Group #3		
*F*–statistic	*P*-value	*H*_0_: equal mean among income classes
1,165.096,	0.00 ^∗∗∗^	not accepted
Observations: 1,643,302		

Legend: ^∗∗∗^ = 1% statistical significance

The null hypothesis cannot be accepted for any of the three demand groups. This means that the superticket expenditure as well as the number of prescriptions is increasing in income. The differences among the income groups may also be due to an age effect (young, healthy individuals may have a lower average income; or retired people may have a pathology exemption and are not considered in our sample) and it would be worth to investigate it further.

Finally, we investigate the demand for prescriptions using the model presented in Eq. (1). The results for the complete sample are presented in Table 6, both for Model #1 (copayment expenditure as dependent variable) and for Model #2 (cost for the regional health care regional system as dependent variable).^8^ As expected, consumption has a positive and statistically significant impact on copayment expenditure (+3.805). On average, the copayment paid is increasing with income (the no-income class is the baseline); this confirms that the system is slightly progressive as shown by the Kakwani index. Female patients pay more than male (+3.701); copayment expenditure increases with the patient’s age (+0.236) and is lower the higher the number of children (-0.285). This may be due to the low health care demand of young people living with the family. The copayment expenditure is higher if there are family members with disability (+2.622), but it is lower if the spouse has no income (-0.662). Finally, self-employed have lower copayment than the baseline income group (-0.662), while employed and retired have higher copayment expenditures (respectively +2.632 and +1.694). As shown in Table 6 we have controlled for the ASL fixed effects and for some demographic characteristics of the prescription’s payer, i.e., whether it is single or married, divorced, widower, etc. The results for the regional health care cost are similar; for this reason they are not discussed.

**Table 6:** Determinants of individual copayment and cost-of-service

	Dependent variable	
Independent variables	Copayment (Model #1)	Cost (Model #2)
Number of prescriptions	3.805^c^	9.055^c^
Female	3.701^c^	–6.507^c^
Age	0.236^c^	0.946^c^
Number of children	–0.285^c^	–2.190^c^
Family members with disability	2.622^c^	4.915^a^
Dependent spouse	–0.662^c^	–6.176^c^
Self Employed	–1.194^c^	0.817
Employed	2.632^c^	6.631^c^
Retired	1.694^c^	–1.715^a^
Income classes		
<8,000	–0.546	–3.672
8,001−15,000	2.768^c^	3.536
15,001−24,000	3.991^c^	9.651^a^
24,001−35,000	5.613^c^	12.61^c^
35,001−55,000	8.880^c^	19.70^c^
55,001−75,000	7.062^c^	10.46^b^
>75,001	–0.48	–4.908
Demand groups		
Group #1	–36.22^c^	–32.09^c^
Group #2	–36.59^c^	–61.42^c^
Interaction income classes-demand groups		
<8,000× Group #1	–0.535	–0.891
<8,000× Group #2	–1.068	3.676
8,001−15,000× Group #1	–6.199^c^	–13.24^b^
8,001−15,000× Group #2	–6.126^c^	–11.03
15,001−24,000× Group #1	–6.092^c^	–14.79^c^
15,001−24,000× Group #2	–8.025^c^	–19.92
24,001−35,000× Group #1	–8.092^c^	–17.79^c^
24,001−35,000× Group #2	–9.537^c^	–24.93^a^
35,001−55,000× Group #1	–10.06^c^	-22.44^c^
35,001−55,000× Group #2	–12.23^c^	–32.42^b^
55,001−75,000× Group #1	–9.340^c^	–16.85^c^
55,001−75,000× Group #2	–9.743^c^	–22.48^a^
>75,001× Group #1	–5.991^c^	–10.16^a^
>75,001× Group #2	–3.142	–8.194
ASL dummies	included	included
Marital status dummies	included	included
Constant	40.12^c^	40.48^c^
Observations	2,301,571	2,301,571
R-squared	0.607	0.166
BIC	24,620,754.3	32,831,101.4

Legend: ^a^ 10% significance level; ^b^ 5%; ^c^ 1%

**Fig. 3:**
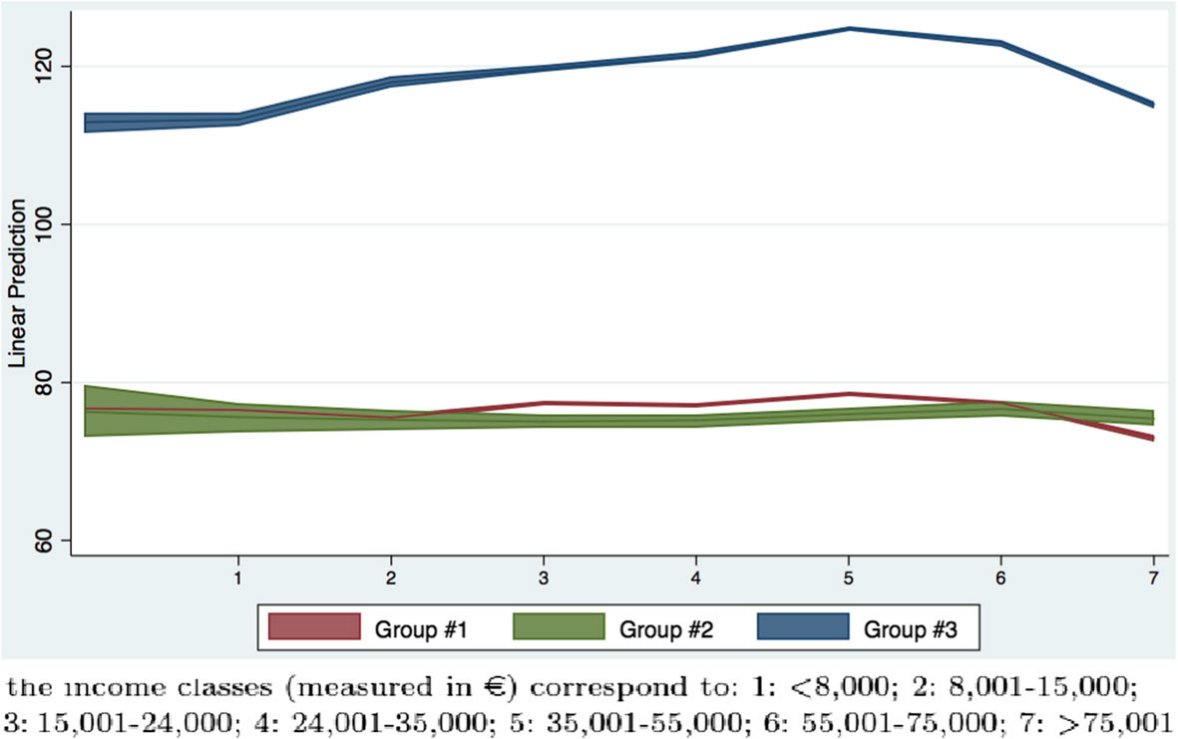
Marginal effect of income classes on superticket

From Table 6 we can obtain the marginal effects of income for the three demand groups (computed at the sample mean of the other variables). They are shown in Fig. 3. The income classes are reported on the horizontal axis while the predicted superticket payment is on the vertical axis. High users (Group #3) pay higher copayments, increasing with income, while the other two groups have similar trends, both in terms of copayment expenditure and of variation with income. The blue line represents the average predicted copayment expenditure for high users (Group #3). The expenditure increases for the first five income classes and then decreases. This result confirms that low income classes have a lower expenditure for patients belonging to Group #3. The decrease in copayment expenditure for high income classes may be due to different factors, e.g., a supplementary health care insurance. For the other two demand groups there does not seem to be the same pattern, with the only exception of the decrease in copayment expenditure for high income classes in Group #1.

This is a confirmation that income seems to be an important determinant of demand for high users, and that the superticket, by rising the price, may have increased this difference even further.

**Fig. 4:**
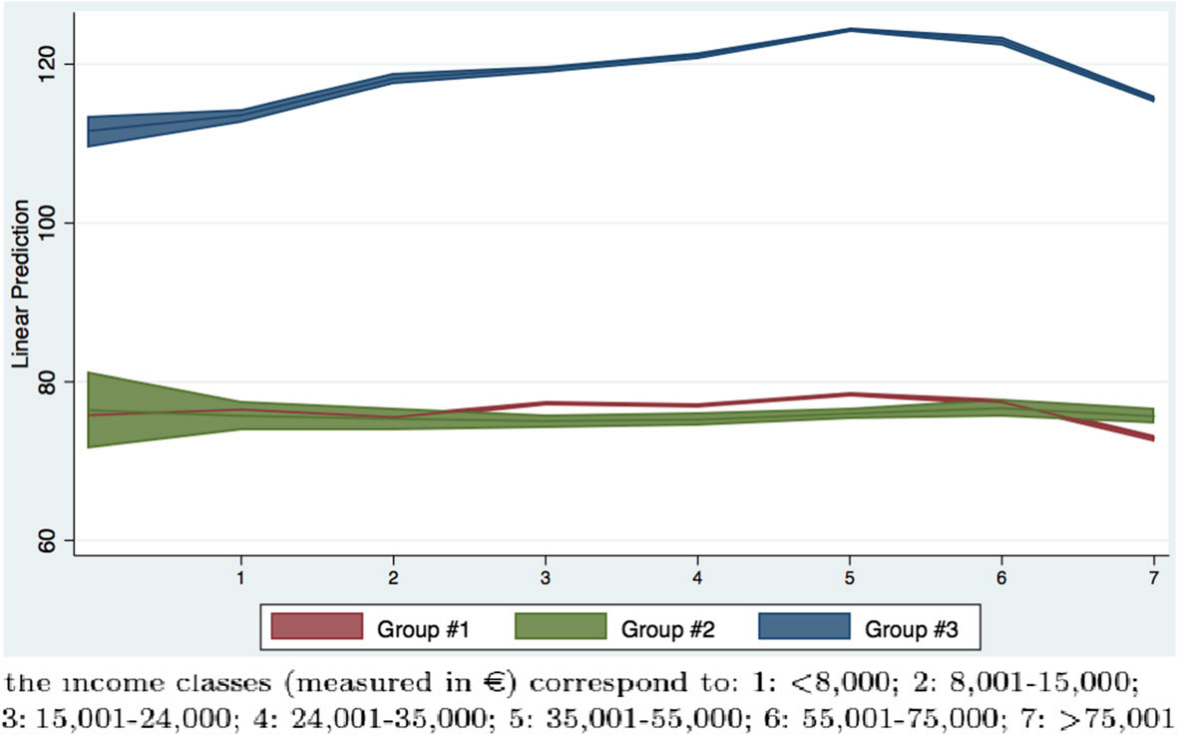
Marginal effects of income classes on superticket when self-employed are included

**Fig. 5:**
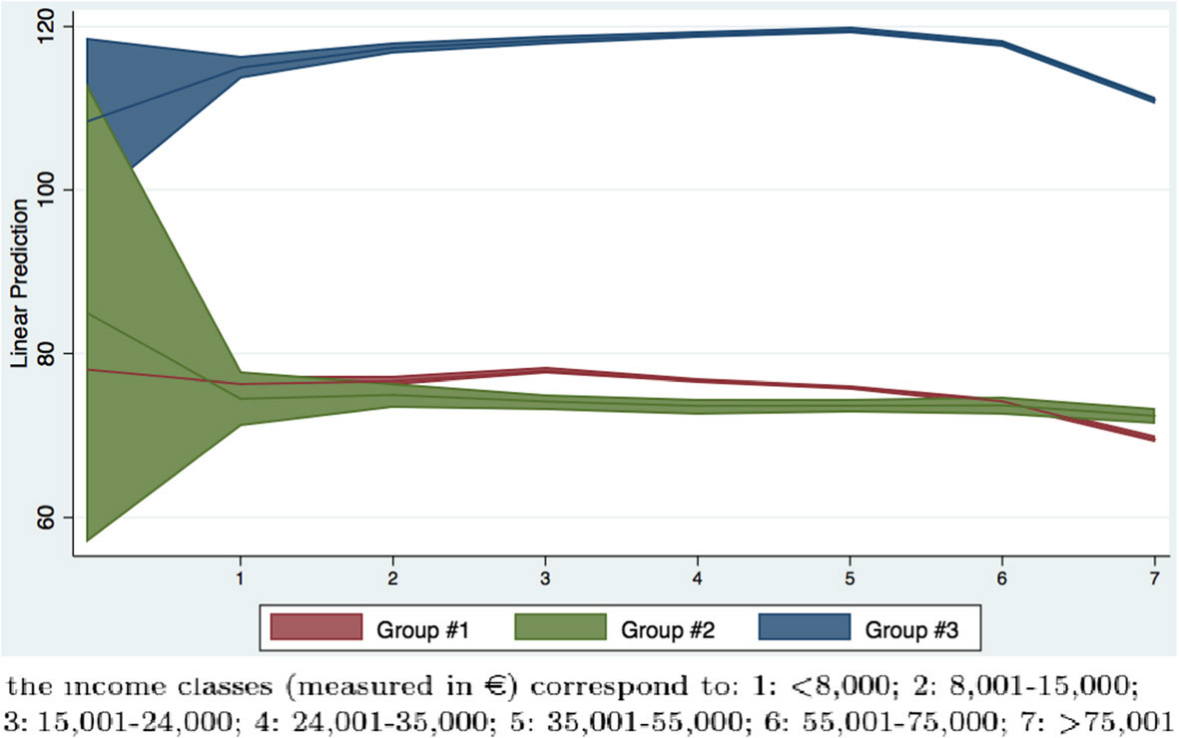
Marginal effects of income classes on superticket. Self-employed and employed sub-sample

A shortcoming of our fiscal data is that income may be underestimated due to tax evasion. This is more frequent for self-employed. For this reason, we have performed a sensitivity analysis by re-estimating Model #1 including a dummy variable for self-employed and its interaction with demand groups and income classes shown in Tables 7 and 8. Figures 4 and 5 present the same marginal effects of income in the sensitivity analysis. They are similar to those presented in Fig. 3; hence, even if we take tax evasion into account we obtain the same evidence (i.e., the superticket may be associated with a significant difference in expenditure especially for high user patients).

**Table 7:** Sensitivity analysis: including a dummy only for self-employed interacted with income classes and demand groups

Variables	Copayment (Model #1)	Cost (Model #2)
Number of prescriptions	3.805***	9.052***
Female	3.592***	–6.630***
Age	0.222***	0.792***
Number of children	–0.286***	–1.746***
Family members with disability	2.674***	5.387*
Dependent spouse	–0.444***	–5.422***
Self Employed	2.489	–0.487
Income classes		
8,000	2.641*	-1.856
8,001−15,000	7.573***	7.664
15,001−24,000	9.287***	14.36*
24,001−35,000	11.18***	18.55**
35,001−55,000	14.76***	26.01***
55,001−75,000	12.74***	16.41*
>75,001	5.228***	1.454
Demand groups		
Group #1	–35.69***	–32.45***
Group #2	–34.74***	–59.10**
Interaction income classes-demand groups		
8,000 # Group #1	–1.688	–5.578
8,000 # Group #2	–3.386	–0.212
8,001−15,000 # Group #1	–7.694***	–15.86
8,001−15,000 # Group #2	–8.731**	–14.29
15,001−24,000 # Group #1	–7.252***	–15.36
15,001−24,000 # Group #2	–10.53***	–22.78
24,001−35,000 # Group #1	–9.409***	–18.91*
24,001−35,000 # Group #2	–12.16***	–28.55
35,001−55,000 # Group #1	–11.39***	–23.16**
35,001−55,000 # Group #2	–14.90***	–35.84
55,001−75,000 # Group #1	–10.52***	–17.76*
55,001−75,000 # Group #2	–12.02***	–24.82
>75,001 # Group #1	–7.876***	–13.06
>75,001 # Group #2	–5.68	–10.94
Interaction income classes-self employed		
8,000 # Self Employed	–3.786*	–3.611
8,001−15,000 # Self Employed	–5.629***	–4.236
15,001−24,000 # Self Employed	–8.117***	–5.236
24,001−35,000 # Self Employed	–9.487***	–11.69
35,001−55,000 # Self Employed	–11.13***	–13.32
55,001−75,000 # Self Employed	–8.184***	–6.413
>75,001 # Self Employed	–6.888***	–4.844
Interaction demand groups-self employed		
Group #1 # Self Employed	–0.796	0.195
Group #2 # Self Employed	–2.629	–3.847
Interaction income classes-demand groups-self employed		
8,000 # Group #1 # Self Employed	2.39	12.91
8,000 # Group #2 # Self Employed	3.949	7.438
8,001−15,000 # Group #1 # Self Employed	4.813**	11.95
8,001−15,000 # Group #2 # Self Employed	6.03	7.423
15,001−24,000 # Group #1 # Self Employed	5.258**	5.518
15,001−24,000 # Group #2 # Self Employed	7.415	6.923
24,001−35,000 # Group #1 # Self Employed	6.387***	9.65
24,001−35,000 # Group #2 # Self Employed	8.242*	12.58
35,001−55,000 # Group #1 # Self Employed	7.309***	9.288
35,001−55,000 # Group #2 # Self Employed	9.505*	14.1
55,001−75,000 # Group #1 # Self Employed	4.911**	8.113
55,001−75,000 # Group #2 # Self Employed	5.503	4.98
>75,001 # Group #1 # Self Employed	5.616***	11.57
>75,001 # Group #2 # Self Employed	5.087	4.929
ASL dummies	included	included
Marital status dummies	included	included
Constant	38.15***	47.79***
Observations	2,301,571	2,301,571
R-squared	0.607	0.166
BIC	24,620,686.1	32,831,464.5

Legend: * 10% significance level; ** 5%; *** 1%

**Table 8:** Sensitivity analysis: self-employed dummy. Self-employed and employed subsample

Variables	Copayment (Model #1)	Cost (Model #2)
Number of prescriptions	3.705***	8.874***
Female	4.517***	–8.412***
Age	0.386***	1.175***
Number of children	–0.00863	–1.698***
Family members with disability	3.171***	6.652**
Dependent spouse	–1.598***	–8.886***
Self Employed	6.185	6.629
Income classes		
8,000	9.087	7.45
8,001−15,000	11.41	15.39
15,001−24,000	12.95	23.81
24,001−35,000	13.86*	24.81
35,001−55,000	14.17*	26.16
55,001−75,000	11.85	14.4
>75,001	4.776	–1.135
Demand groups		
Group #1	–28.20***	–30.86
Group #2	–18.68	–47.81
Interaction income classes-demand groups		
8,000 # Group #1	–11.07	–9.335
8,000 # Group #2	-22.46	-9.417
8,001−15,000 # Group #1	–12.66	–12.19
8,001−15,000 # Group #2	–23.89	–19.9
15,001−24,000 # Group #1	–12.49	–13.5
15,001−24,000 # Group #2	–25.99	–32.26
24,001−35,000 # Group #1	–14.62	–16.5
24,001−35,000 # Group #2	–27.4	–37.17
35,001−55,000 # Group #1	–16.11*	–21.23
35,001−55,000 # Group #2	–27.75	–40.61
55,001−75,000 # Group #1	–15.81	–15.64
55,001−75,000 #	–25.24	–28.98
>75,001 # Group #1	–13.87	–11.67
>75,001 # Group #2	–19.66	–15.39
Interaction income classes-self employed		
8,000 # Self Employed	–10.62	–13.94
8,001−15,000 # Self Employed	–10.21	–14.02
15,001−24,000 # Self Employed	–12.8	–17.72
24,001−35,000 # Self Employed	–13.53*	–21.94
35,001−55,000 # Self Employed	–12.68	–19.61
55,001−75,000 # Self Employed	–9.665	–11.28
>75,001 # Self Employed	–8.995	–9.57
Interaction demand groups-self employed		
Group #1 # Self Employed	–9.045	–3.248
Group #2# Self Employed	–20.12	–17.39
Interaction income classes-demand groups-self employed		
8,000 # Group #1 # Self Employed	11.78	16.78
8,000 # Group #2# Self Employed	23.15	16.86
8,001−15,000 # Group #1 # Self Employed	9.68	8.141
8,001−15,000 # Group #2# Self Employed	21	12.45
15,001−24,000 # Group #1 # Self Employed	10.31	3.395
15,001−24,000 # Group #2# Self Employed	22.55	15.59
24,001−35,000 # Group #1 # Self Employed	11.44	7.053
24,001−35,000 # Group #2# Self Employed	23.14	20.38
35,001−55,000 # Group #1 # Self Employed	12.1	7.858
35,001−55,000 # Group #2# Self Employed	22.54	19.6
55,001−75,000 # Group #1 # Self Employed	10.26	6.537
55,001−75,000 # Group #2# Self Employed	18.97	10.18
>75,001 # Group #1 # Self Employed	11.57	10.58
>75,001 # Group #2# Self Employed	19.14	10.25
ASL dummies	included	included
Marital status dummies	included	included
Constant	29.53***	29.71
Observations	1,688,020	1,688,020
R-squared	0.599	0.162
BIC	17,951,547.9	23,988,370.4

Legend: * 10% significance level; ** 5%; *** 1%

## Discussion

The revenue from the superticket in 2012 was in line with what was expected: the 135 million reduction in the grant from Central Government was matched by an extra revenue of around 132 million from the superticket. However, the introduction of the extra charge means that outpatient treatments whose reimbursement is below €51 are charged more than what reimbursed to the provider (see Table 1). In year 2012 this produced a net revenue equal to €46,759,557; this income source may drop in the years to come: if patients become aware of this extra payment, they may start looking for cheaper alternatives in the private market. Indeed, private labs are starting to attract patients out of the public health care system by advertising lower prices than the copayment (see, for example, http://novolabs.it/index.php/tariffe/ssn-e-ticket). The non linear schedule chosen by Lombardy means that prescriptions in the €0-36 cost range cost to patients less than under the uniform system; users in this class are somehow the gainer of this reform. Our results shows that about 70% of non exempt prescriptions falls in this range and that in general they form a more consistent share of the prescriptions demanded by low income groups, but this result coexists with evidences pointing towards differences in health care expenditure caused by the superticket. This is particularly relevant for the high-users, as shown by our analysis.

## Conclusions

The increase in the out of pocket payment due to the introduction of the superticket raises questions about the distribution of the burden among patients and the barriers to access that it may have created. From the revenue side, in the short run, the policy is sustainable, but competition from private providers casts some doubts on long-run perspectives.

On the distribution side, the “paternalistic goods” nature of health care services [6, 8] means that there are two dimensions on which redistribution should be evaluated: (1) the health status measuring the need for care and income, (2) the income effects in the access to health care. Our data do not allow to study the first dimension since the only available health-related information is exemption for pathology. This issue should be furtherly investigated in future research where this dataset might be matched with health status variables in order to assess whether the superticket may also create barriers to the access to health care services.

## Endnotes

^1^ The EU-SILC (Statistics on Income and Living Conditions) is one of the main source of information on social and economic conditions in Member States. It is based on survey data and it is the most important source available in Europe for individual income distributions.

^2^ The difference is due to the very small number of individuals that have only tax with holding income declarations; the latter do not report identifiers for other family members. Therefore they are treated as individuals with independent income, and this slightly increases the left tie frequency of the distribution of our income data in comparison to those of EU-SILC.

^3^ This occurrence is rather limited: according to official statistics unemployment in Lombardy increased from 5.5% in 2010 to 7.4% in 2012; which means an increase in unemployed people by 95,000 units. Since our dataset covers 5,805,177 individuals having declared an income in 2010 and receiving at least one treatment in 2012, the maximum possible incidence of this temporal asymmetry is rather small (less than 2%).

^4^ A fraction of individuals had to be dropped from the analysis because it was impossible to get a match for their income, mainly due to unemployment conditions or administrative residence outside the region.

^5^ These are the general principles; the system foresees exceptions and special cases. For a more specific description see [7, 10].

^6^ The standard ANOVA test requires equal variance among the different income classes. If this is not fulfilled, as in our case, it is possible to apply the simulated ANOVA, which simulates 1000 replications of the standard ANOVA test and computes how many times the *p*-value of the test is higher than that of the standard ANOVA. If the *p*-value of the simulated ANOVA test is lower than that of the standard test the results obtained with the latter are not distorted.

^7^ It is possible to argue that this amount represents a potential loss for the regional budget if patients, becoming aware that they pay more than the prescription costs, decide to swap their demand to the private sector, where they may pay less.

^8^ To save space standard errors are not reported.

## Abbreviation

EU-SILC: European - Statistics on income and living conditions

## References

[1] Atella V, Peracchi F, Depalo D, Rossetti C (2006). Drug compliance, co-payment and health outcomes: evidence from a panel of italian patients. Health Econ.

[2] Fiorio CV, Siciliani L (2010). Co-payments and the demand for pharmaceuticals: Evidence from Italy. Econ Model.

[3] Kakwani NC (1977). Measurement of tax progressivity: An international comparison. Econ J.

[4] Kiil A, Houlberg K (2014). How does copayment for health care services affect demand, health and redistribution? a systematic review of the empirical evidence from 1990 to 2011. Eur J Health Econ.

[5] Levaggi L, Levaggi R, Brosio G, Muraro G (2006). Optimal copayment strategies in a public health care system. Il finanziamento del settore pubblico.

[6] Levaggi L, Levaggi R (2011). Welfare properties of restrictions to health care based on cost effectiveness. Health Econ.

[7] Mastrobuono I, Visconti G, Sorbara D, Labate G (2012). L’evoluzione della compartecipazione alla spesa sanitaria in europa: possibili scenari evolutivi e proposte di riorganizzazione in italia. Igiene e Sanita’ Pubblica.

[8] Schnellenbach J (2012). Nudges and norms: On the political economy of soft paternalism. Eur J Polit Econ.

[9] Sinnott SJ, Buckley C, O’Riordan D, Bradley C, Whelton H (2013). The effect of copayments for prescriptions on adherence to prescription medicines in publicly insured populations; a systematic review and meta-analysis. PLoS ONE.

[10] Thompson S. International profiles of health care systems 2013. Tech. Rep 1717, The Commonwealth Fund. 2013. http://www.commonwealthfund.org/.

[11] Verzillo S, Santoro A, Mezzanzanica M, (forthcoming). Family splitting versus joint taxation: a case-study. J Anal Inst Econ - Econ Politica. doi:10.1007/s40888-016-0039-x.

